# Supplementary material for: MitoQ Is Able to Modulate Apoptosis and Inflammation
Source: Int J Mol Sci. 2021 Apr 30;22(9):4753. doi: 10.3390/ijms22094753 (PMC8124358; doi:10.3390/ijms22094753)
Supplement: Supplementary file 1 [file ijms-22-04753-s001.zip › ijms-1182966-supplementary/ijms-1182966-proofed-supplementary.pdf]

Supplementary Table S1. Pro-inflammatory cytokines profile of DAOY cells after 24 and 48 hours of treatment.

Cytokine (pg/ml) \ Stimulus	24 h				48 h			
	**Untr**	**Lova**	**MitoQ+ Lova**	**MitoQ**	**Untr**	**Lova**	**MitoQ+ Lova**	**MitoQ**
**IL-1b**	11.55 ± 1.28	15.25 ± 0.99	11.46 ± 1.84	14.41 ± 1.23	11.33 ± 0.70	13.64 ± 2.81	8.98 ± 2.26	15.44 ± 0.79
**IL-2**	43.64 ± 2.91	52.43 ± 4.29	39.39 ± 10.17	48.87 ± 8.44	43.93 ± 1.27	48.27 ± 9.85	33.07 ± 6.43	55.29 ± 4.91
**IL-4**	15.26 ± 1.64	19.03 ± 3.67	13.06 ± 2.70	20.93 ± 2.39	17.69 ± 1.04	20.10 ± 3.58	14.38 ± 2.19	22.60 ± 1.37
**IL-17**	29.09 ± 0.96	40.91 ± 4.52	29.60 ± 8.96	40.51 ± 4.26	32.43 ± 1.51	39.95 ± 6.37	28.94 ± 5.38	41.18 ± 4.53
**IL-6**	$10.9 \times 10^3 \pm 90.2 \times 10^1$	$14.0 \times 10^3 \pm 24.3 \times 10^2$	$99.8 \times 10^2 \pm 12.0 \times 10^2$	$97.0 \times 10^2 \pm 15.8 \times 10^2$	$1.16 \times 10^3 \pm 13.8 \times 10^2$	$17.00 \times 10^3 \pm 22.60 \times 10^2$	$76.10 \times 10^2 \pm 14.90 \times 10^2$	$1.98 \times 10^3 \pm 62.00 \times 10^2$
**IL-8**	$17.90 \times 10^3 \pm 35.80 \times 10^1$	$21.80 \times 10^3 \pm 15.50 \times 10^2$	$18.00 \times 10^3 \pm 36.46 \times 10^2$	$22.75 \times 10^3 \pm 11.21 \times 10^2$	$21.57 \times 10^3 \pm 13.12 \times 10^2$	$24.10 \times 10^3 \pm 35.59 \times 10^2$	$18.03 \times 10^3 \pm 36.46 \times 10^2$	$22.82 \times 10^3 \pm 10.72 \times 10^2$
**IFNg**	$63.0 \times 10^1 \pm 11.16 \times 10^1$	$75.87 \times 10^1 \pm 12.03 \times 10^1$	$67.91 \times 10^1 \pm 14.13 \times 10^1$	$85.99 \times 10^1 \pm 92.43$	$86.69 \times 10^1 \pm 79.38$	$95.23 \times 10^1 \pm 18.57 \times 10^1$	$67.91 \times 10^1 \pm 14.13 \times 10^1$	$11.61 \times 10^2 \pm 61.21$
**TNFa**	$23.02 \times 10^1 \pm 72.48$	$18.41 \times 10^1 \pm 70.86$	$17.24 \times 10^1 \pm 56.92$	$14.82 \times 10^1 \pm 67.35$	$31.18 \times 10^1 \pm 23.28$	$36.25 \times 10^1 \pm 87.98$	$22.97 \times 10^1 \pm 25.49$	$39.82 \times 10^1 \pm 33.06$

Cytokines quantification is e×pressed in pg/ml.
